# Supplementary material for: Protrudin and PDZD8 contribute to neuronal integrity by promoting lipid extraction required for endosome maturation
Source: Nat Commun. 2020 Sep 11;11:4576. doi: 10.1038/s41467-020-18413-9 (PMC7486383; doi:10.1038/s41467-020-18413-9)
Supplement: Supplementary file 1 — Supplementary Information [file 41467_2020_18413_MOESM1_ESM.pdf]

Supplementary Information

Protrudin and PDZD8 contribute to neuronal integrity by promoting lipid extraction required for endosome maturation

Michiko Shirane, Mariko Wada, Keiko Morita, Nahoki Hayashi, Reina Kunimatsu, Yuki Matsumoto, Fumiko Matsuzaki, Hirokazu Nakatsumi, Keisuke Ohta, Yasushi Tamura & Keiichi I. Nakayama

Supplementary Text
Supplementary Tables 1 and 2
Supplementary Figure Legends
Supplementary Figures 1–17

Supplementary Text

Autophagy induction, lysosomal activity, and LyLE positioning in HeLa cells coexpressing protrudin and PDZD8

Forced coexpression of protrudin and PDZD8 in HeLa cells by transient transfection induced a small increase in LC3 immunofluorescence per cell (Supplementary Fig. 14a, b), suggestive of an increased extent of autophagy. However, immunofluorescence signals for the EE marker EEA1 (Supplementary Fig. 14c) and the LyLE marker LAMP1 (Supplementary Fig. 14d, e) were also increased in cells overexpressing protrudin and either PDZD8(WT) or PDZD8(ΔSMP), suggesting that the increase in the LC3 signal might reflect disruption of endosomal homeostasis as a result of such overexpression. Similar results were obtained by immunoblot analysis (Supplementary Fig. 15). Lysosomal activity as reflected by the fluorescence intensity of Magic red (cathepsin K indicator) was also increased in association with LyLE enlargement in cells overexpressing protrudin and PDZD8 (Supplementary Fig. 16).

We also examined the distribution of LyLEs in HeLa cells expressing HA-protrudin and PDZD8-FLAG by immunofluorescence analysis with antibodies to LAMP1 and to FLAG. LAMP1 signals showed marked aggregation around the large MVB-like structures in cells expressing protrudin and PDZD8(WT) (Supplementary Fig. 14e). In cells expressing protrudin and PDZD8(ΔSMP), however, no formation of the MVB-like structures was apparent and the LAMP1 signals were dispersed around the nucleus (Supplementary Fig. 14e). On the other hand, depletion of protrudin in HeLa cells resulted in abnormal LyLE positioning (Supplementary Fig. 17), consistent with previous observations[8]. In contrast, PDZD8 depletion did not affect LyLE positioning. We therefore concluded that, unlike protrudin, PDZD8 does not contribute to LyLE positioning.

Supplementary Table 1 | List of Stealth siRNAs

rat protrudin

Name	Sequence
r-protrudin-1	5'-CCCTACCAAGTCAACAGCGTTTGAT-3'
r-protrudin-2	5'-TCAACATCTTGTTCCTCACTCTGAA-3'
r-protrudin-3	5'-TACCTGCGAGTCAGCTTACCGTGT-3'

rat PDZD8

Name	Sequence
r-PDZD8-1	5'-GGGCCGGCTTAAAGTTACATTGCTA-3'
r-PDZD8-2	5'-CAGTCCCAAACGTACTCCAACAACA-3'
r-PDZD8-3	5'-GAGGTGGCTTTAGGATGCCTAGCTA-3'

human protrudin

Name	Sequence
h-protrudin-1	5'-GAGCUGAUGCGGAGGAAGUAUCAUA-3'
h-protrudin-2	5'-AGGAUGCAGGUGAUGGUGUUCGAUA-3'
h-protrudin-3	5'-AUGCCCUGUACUCAGACACAACUCG-3'

human PDZD8

Name	Sequence
h-PDZD8-1	5'-GGAGUUCUAUUAAGACGGUUGAAUU-3'
h-PDZD8-2	5'-CCAUUUGGUUGAAGAAGUUUCUGUU-3'
h-PDZD8-3	5'-UGCAGUUAAAGAAAUUGGUCGUGAU-3'

mouse protrudin

Name	Sequence
m-protrudin-1	5'-AUCAAACGCUGUUGACUUGGUAGGG-3'
m-protrudin-2	5'-UUCAGAGUGAGGAACAAGAUGUUGA-3'
m-protrudin-3	5'-UUAUCAAGGCACCCACGGAGUACCA-3'

mouse PDZD8

Name	Sequence
m-PDZD8-1	5'-CCAUAUUCAACAAUGGGCACUUACU-3'
m-PDZD8-2	5'-AAGGCCGGCUUAAAGUUACAUUGUU-3'
m-PDZD8-3	5'-UUCGAAUUCAGAAUCUAGGUCUCUG-3'

Supplementary Table 2 | List of primers for PCR

rat protrudin

Name	Sequence
forward	5'-ACAGAGGACCTCACGCCAGGC-3'
reverse	5'-TCATTTGCTCAAGGTCTGATTAC-3'

rat PDZD8

Name	Sequence
forward	5'-ACGTGGATCTAGTGTTCGGCA-3'
reverse	5'-GGTCCTCCAGGAAGGAGAAGA-3'

rat GAPDH

Name	Sequence
forward	5'-AAGGCTGAGAATGGGAAGCTG-3'
reverse	5'-GGAGATGATGACCCTTTTGGC-3'

human protrudin

Name	Sequence
forward	5'-CTCTGCTGGGTTCTCACCCTT-3'
reverse	5'-TGGTGGTGGAGGACTCTCAAA-3'

human PDZD8

Name	Sequence
forward	5'-AAGACCCGCTGATCGACTTC-3'
reverse	5'-CTTGCAAGGTCTGGTATGGAAA-3'

human GAPDH

Name	Sequence
forward	5'-GTCAGTGGTGGACCTGACCTG-3'
reverse	5'-AAAGTGGTCGTTGAGGGCAAT-3'

mouse protrudin

Name	Sequence
forward	5'-TACCAAGTCAGCAGCGTTTG-3'
reverse	5'-GCAAGTCAGCAAGGAACACA-3'

mouse PDZD8

Name	Sequence
forward	5'-TCAACTGATGGGTATGCTGG-3'

reverse	5'-ATAGCAATGAGCCGATCTCC-3'

mouse GAPDH

Name	Sequence
forward	5'-CATGGCCTTCCGTGTTCCTA-3'
reverse	5'-GCGGCACGTCAGATCCA-3'

Supplementary Figure Legends

Supplementary Fig. 1. Validation of protrudin-PDZD8 binding. Extracts of HEK293T cells transfected with plasmids encoding FLAG-protrudin or PDZD8-Myc, or with corresponding control vectors for FLAG-tagged proteasome subunit S4 or Cul1-Myc, were subjected to immunoprecipitation (IP) with antibodies to FLAG (**a**) or with either antibodies to Myc or control IgG (**b**). The resulting precipitates, as well as 8% of the input material for immunoprecipitation, were then subjected to immunoblot (IB) analysis with antibodies to FLAG, to Myc, and to HSP90 (loading control). All epitope-labeled proteins were mouse. We repeated all experiments at least three times independently with similar results.

Supplementary Fig. 2. Identification of the region of protrudin responsible for interaction with PDZD8. FLAG-tagged full-length protrudin or its mutants shown in Figure 1d were expressed in HEK293T cells together with HA-tagged mouse PDZD8. Cell extracts were subjected to immunoprecipitation with antibodies to FLAG, and the resulting precipitates, as well as 5% of the input for immunoprecipitation, were subjected to immunoblot analysis with antibodies to FLAG, to HA, and to HSP90. The asterisk indicates nonspecific signals. We repeated all experiments at least three times independently with similar results.

Supplementary Fig. 3. Identification of the regions of PDZD8 responsible for interaction with protrudin. Full-length PDZD8 or its mutants shown in Figure 1e fused to the Myc (**a**) or FLAG (**b**) tags were expressed in HEK293T cells together with FLAG- or HA-tagged protrudin, respectively. Cell extracts were subjected to immunoprecipitation with antibodies to Myc (**a**) or to FLAG (**b**), and the resulting precipitates, as well as a portion of the input for immunoprecipitation, were subjected to immunoblot analysis with antibodies to FLAG, to Myc, to HA, and to HSP90. We repeated all experiments at least three times independently with similar results.

Supplementary Fig. 4. Protrudin and PDZD8 mutually stabilize each other. Protein extracts prepared from the brain of WT (+/+) or PDZD8-deficient (–/–) mice (**a**), or from that of WT (+/+) or protrudin-deficient (–/–) mice (**b**), were subjected to immunoblot analysis with antibodies to protrudin, to PDZD8, and to either calnexin or

TMEM55B (loading control). Filled and open arrowheads indicate bands corresponding to protrudin in PDZD8$^{-/-}$ mice and to PDZD8 in protrudin$^{-/-}$ mice, respectively. The asterisk indicates nonspecific signals. The animals studied were different from those examined in Figure 1g and 1h. We repeated all experiments at least three times independently with similar results.

Supplementary Fig. 5. RNA interference–mediated depletion of protrudin and PDZD8 mRNAs in PC12 cells. PC12 cells transfected with protrudin, PDZD8, or control siRNAs were subjected to quantitative reverse transcription and polymerase chain reaction (qRT-PCR) analysis of the targeted mRNAs. Data are means + SD from three independent experiments.

Supplementary Fig. 6. Characterization of the TM domain of PDZD8 with a TM deletion mutant. HeLa cells transfected with expression vectors for WT (**a**) or ΔTM mutant (**b**) forms of mouse PDZD8 tagged with the FLAG epitope were subjected to immunofluorescence analysis with antibodies to FLAG (green) and with those (red) to calreticulin (ER marker) or to Tom20 (mitochondrial marker). Nuclei in the merged images were also stained with Hoechst 33258 (blue). Scale bars, 10 μm. We repeated all experiments at least three times independently with similar results.

Supplementary Fig. 7. Characterization of the TM domain of PDZD8 with chimeric mutants. (**a**, **b**) HeLa cells transfected with expression vectors for FLAG-tagged CYP-PDZD8 (**a**) or Tom20-PDZD8 (**b**) mutant proteins shown in Figure 1f were subjected to immunofluorescence analysis with antibodies to FLAG (green) and with those (red) to calreticulin or to Tom20. Nuclei in the merged images were also stained with Hoechst 33258 (blue). Scale bars, 10 μm. Enlarged images are also shown on the right. Scale bars, 1 μm. (**c**) FLAG-tagged WT or the indicated mutant versions of PDZD8 were expressed in HEK293T cells together with HA-tagged mouse protrudin or the head domain of KIF5 (positive control for protrudin binding protein). Cell extracts were subjected to immunoprecipitation with antibodies to FLAG, and the resulting precipitates, as well as 1% of the input for immunoprecipitation, were subjected to immunoblot analysis with antibodies to HA, to FLAG, and to HSP90. We repeated all experiments at least three times independently with similar results.

Supplementary Fig. 8. Verification of split-GFP assays. (**a**, **b**) HeLa cells transfected with expression vectors for split-GFP fragments—ERj1-GFP(1–10) and either LAMP1-GFP(11) (**a**) or Tom70-GFP(11) (**b**)—were stained with antibodies to the LyLE marker LAMP1 (left) or to the mitochondrial marker Tom20 (right) and imaged by confocal fluorescence microscopy. Scale bars, 10 µm. (**c**) HeLa cells transfected with expression vectors for split-GFP fragments—ERj1-GFP(1–10) and LAMP1-GFP(11)—and for mCherry were imaged by confocal fluorescence microscopy. Fluorescence intensity values were measured by surrounding each cell with a region of interest (ROI) tool and were calculated as shown in Figure 2d. We repeated all experiments at least three times independently with similar results.

Supplementary Fig. 9. Expression level of protrudin and PDZD8 mRNA. (**a**, **b**) HeLa cells transfected with control, protrudin (**a**), or PDZD8 (**b**) siRNAs were subjected to RT and real-time PCR analysis of the targeted mRNAs. Data represent relative mRNA abundance and are means + SD from three independent experiments. (**c**–**f**) HeLa cells infected with retroviruses (**c**, **d**), or subjected to transient transfection with expression vectors (**e**, **f**), for mouse protrudin, mouse PDZD8, or both proteins were subjected to RT and real-time PCR analysis of the endogenous (Endo) and exogenous (Exo) protrudin and PDZD8 mRNAs. (**g**, **h**) HeLa cells or the mouse brain cortex (Br-Cx) or midbrain (Br-Mid) were subjected to RT and real-time PCR analysis of protrudin (**g**) or PDZD8 (**h**) mRNAs. Data in (**c**) to (**h**) represent absolute quantity (10^6 copies/µg of RNA) and are means + SD from three independent experiments.

Supplementary Fig. 10. Velocity of lipid extraction by PDZD8 mutants. (**a**–**c**) Procedure for calculation of the velocity of PS extraction by PDZD8(ΔTM). The fluorescence intensity for 500 nM NBD-PS in the presence of 10 nM GST (**a**) or GST-PDZD8(ΔTM) (**b**), or the value for GST-PDZD8(ΔTM) minus that for GST (**c**), is shown. The total fluorescence value for NBD-PS was determined by the addition of 0.05% Triton X-100 at 310 s after the onset of the reaction. The amount of lipid extracted (nM lipid) = [fluorescence value for GST-PDZD8(ΔTM) – fluorescence value for GST] × 500 nM/fluorescence value after the addition of Triton X-100. The initial velocity of lipid extraction (nM lipid/s) = amount of lipid extracted (nM lipid) during

the initial 10 s/10 s. (**d**) Summary of the initial velocity for lipid extraction by the indicated PDZD8 mutants shown in Figures 3e, 3h, 4c, 4f, and 5g.

Supplementary Fig. 11. Lipid binding ability of PDZD8(C1) mutant. Recombinant GST or GST-PDZD8(C1) was incubated with Membrane Lipid Strips (a different lot from that used in Figure 5b) (**a**) or with cutom lipid strips (**b**), after which binding of the proteins to lipid spots on the strips was probed with antibodies to GST and immunoblot reagents. The lipids on the strips are listed below. We repeated all experiments at least three times independently with similar results.

Supplementary Fig. 12. No ALV formation by stable expression of protrudin and PDZD8. HeLa cells infected with retroviruses for mouse protrudin and PDZD8 were stained with antibodies to calreticulin (**a**) or to LAMP1 (**b**) and then imaged by confocal fluorescence microscopy. Merged fluorescence and differential interference contrast (DIC) images are also shown. Scale bars, 10 μm. Note the absence of vacuoles. We repeated all experiments at least three times independently with similar results.

Supplementary Fig. 13. No enlarged LyLE by forced coexpression of protrudin and PDZD8 in neurons. (**a**) Mouse primary cortical neurons transfected with expression vectors for mouse PDZD8 and protrudin were cultured for 9 days in vitro, fixed, stained with antibodies to the neuronal marker Tubb3 and to the LyLE marker LAMP1, and observed with a confocal fluorescence microscope. Scale bars, 10 μm. The boxed regions of the upper panels are shown enlarged below. Scale bars, 1 μm. (**b**) Quantification of the number of LyLEs with a diameter of >1 μm in images as in (**a**). Data are means + SE for four independent cells. n.s., not significant (Student's *t* test). We repeated all experiments at least three times independently with similar results.

Supplementary Fig. 14. Impaired endosomal homeostasis caused by overexpression of protrudin and PDZD8 in HeLa cells. HeLa cells transiently transfected with expression vectors for HA-protrudin as well as WT or ΔSMP mutant forms of PDZD8-FLAG were stained with antibodies to FLAG as well as with those to LC3 (**b**), to EEA1 (**c**), or to LAMP1 (**d**), and the fluorescence intensity of each organelle marker protein per $FLAG^{+}$ cell was determined. Images for (**b**) and (**d**) are

shown in (**a**) and (**e**), respectively. Red dashed traces in (**a**) and (**e**) indicate the outline of individual cells. Enlarged images of LAMP1 immunoreactivity in the boxed regions of the outlined cells in (**e**) are shown below. Scale bars are as indicated. Quantitative data are means + SE for 12 to 13 independent cells (**b**), 22 to 27 independent cells (**c**), and 20 to 24 independent cells (**d**). $*P < 0.05$, $**P < 0.01$, $***P < 0.001$ (Kruskal-Wallis test followed by the Steel-Dwass multiple comparison test). We repeated all experiments at least three times independently with similar results.

Supplementary Fig. 15. Effect on autophagy of altered expression of protrudin and PDZD8. (**a**) HeLa cells transfected with control or PDZD8 siRNAs or with expression vectors for HA-protrudin and PDZD8-FLAG were subjected to immunoblot analysis with antibodies to LC3, to EEA1, to LAMP1, as well as to HSP90 and to α-tubulin (loading controls). (**b**) Quantification of EEA1 and LAMP1 levels in (**a**) as well as of LC3 levels in additional immunoblots. Data for LC3 are means + SE for three or four independent experiments. $*P < 0.05$ (Kruskal-Wallis test followed by the Steel-Dwass multiple comparison test). We repeated all experiments at least three times independently with similar results.

Supplementary Fig. 16. Increased lysosomal activity by overexpression of protrudin and PDZD8. (**a**) HeLa cells transfected with expression vectors for HA-protrudin and PDZD8-FLAG were metabolically labeled with Magic red and imaged by confocal fluorescence microscopy. Merged fluorescence and DIC images are also shown. Scale bars, 10 μm. (**b**) Quantification of Magic red fluorescence intensity per cell in (**a**). Data are means + SE for 23 to 26 independent cells. $***P < 0.001$ (Kruskal-Wallis test followed by the Steel-Dwass multiple comparison test). We repeated all experiments at least three times independently with similar results.

Supplementary Fig. 17. Effect of depletion of protrudin and PDZD8 on intracellular distribution of LyLEs. HeLa cells transfected with control, protrudin, or PDZD8 siRNAs as indicated were deprived of amino acids for 2 h and then cultured in complete medium for 1 h before staining with antibodies to LAMP1 and imaging by confocal fluorescence microscopy. Red dashed traces indicate the outline of individual

cells. Merged fluorescence and DIC images are also shown. Scale bars, 10 μm. We repeated all experiments at least three times independently with similar results.

a

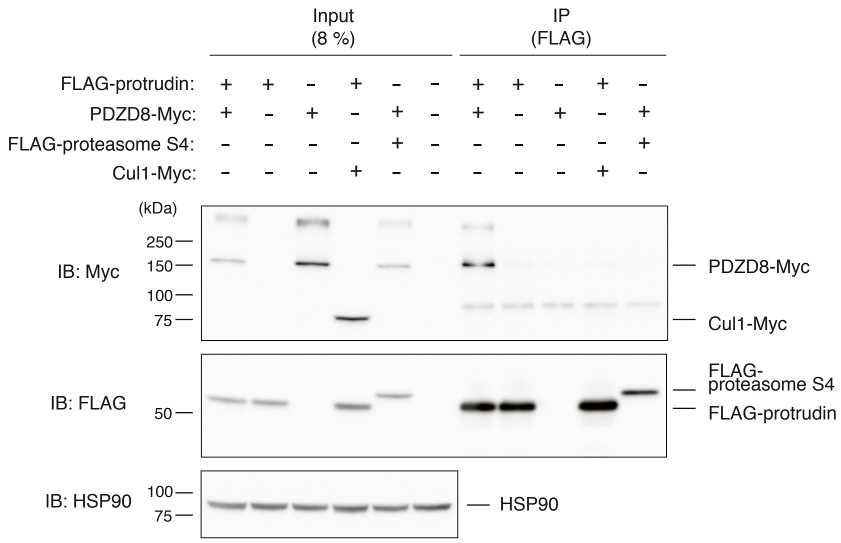

b

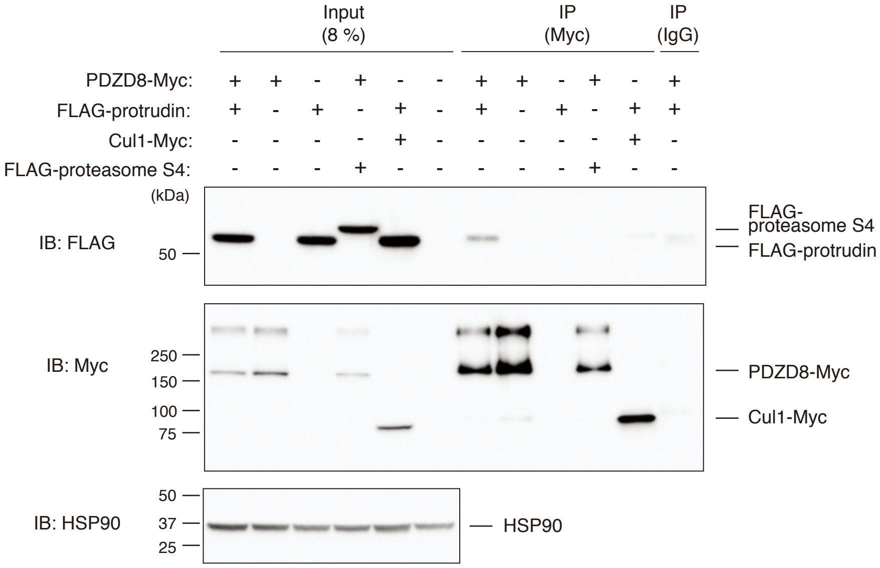

Shirane et al. Figure S1

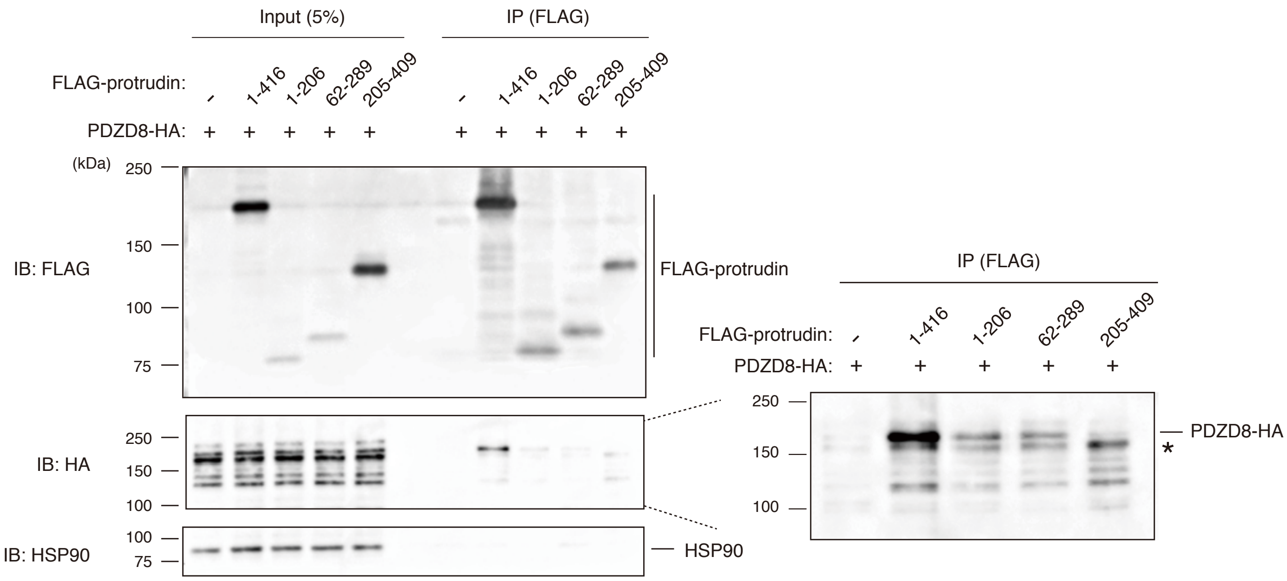

Shirane et al. Figure S2

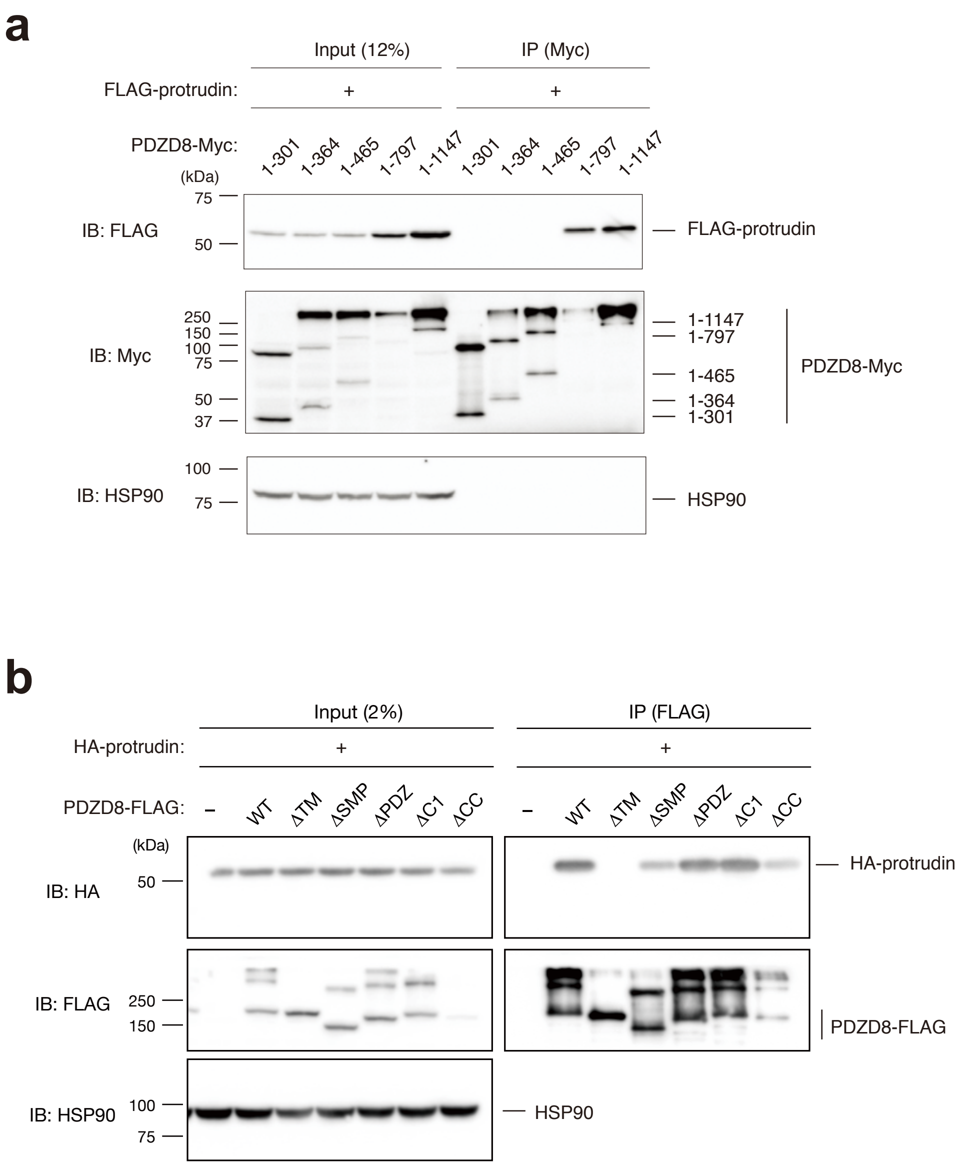

Shirane et al. Figure S3

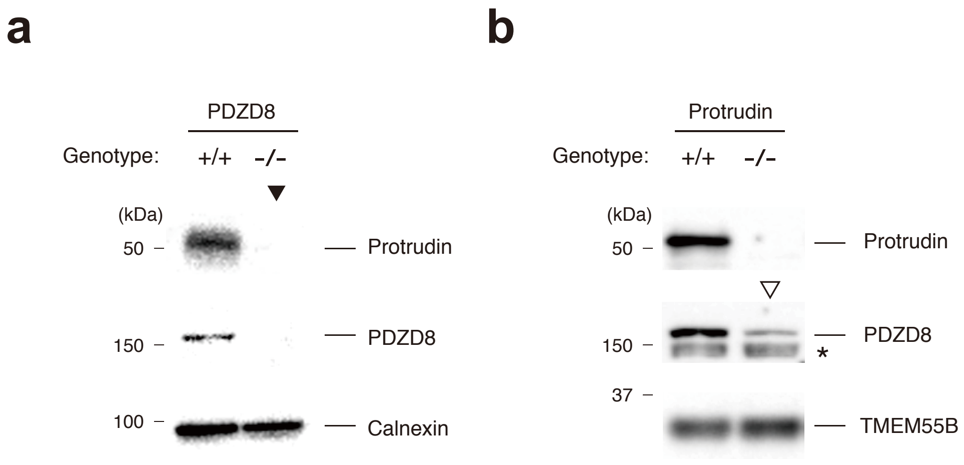

Shirane et al. Figure S4

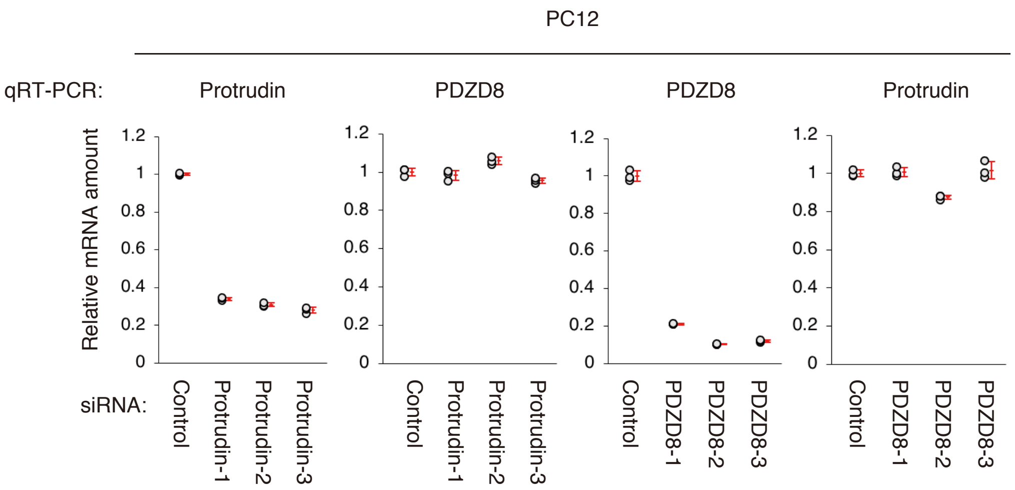

Shirane et al. Figure S5

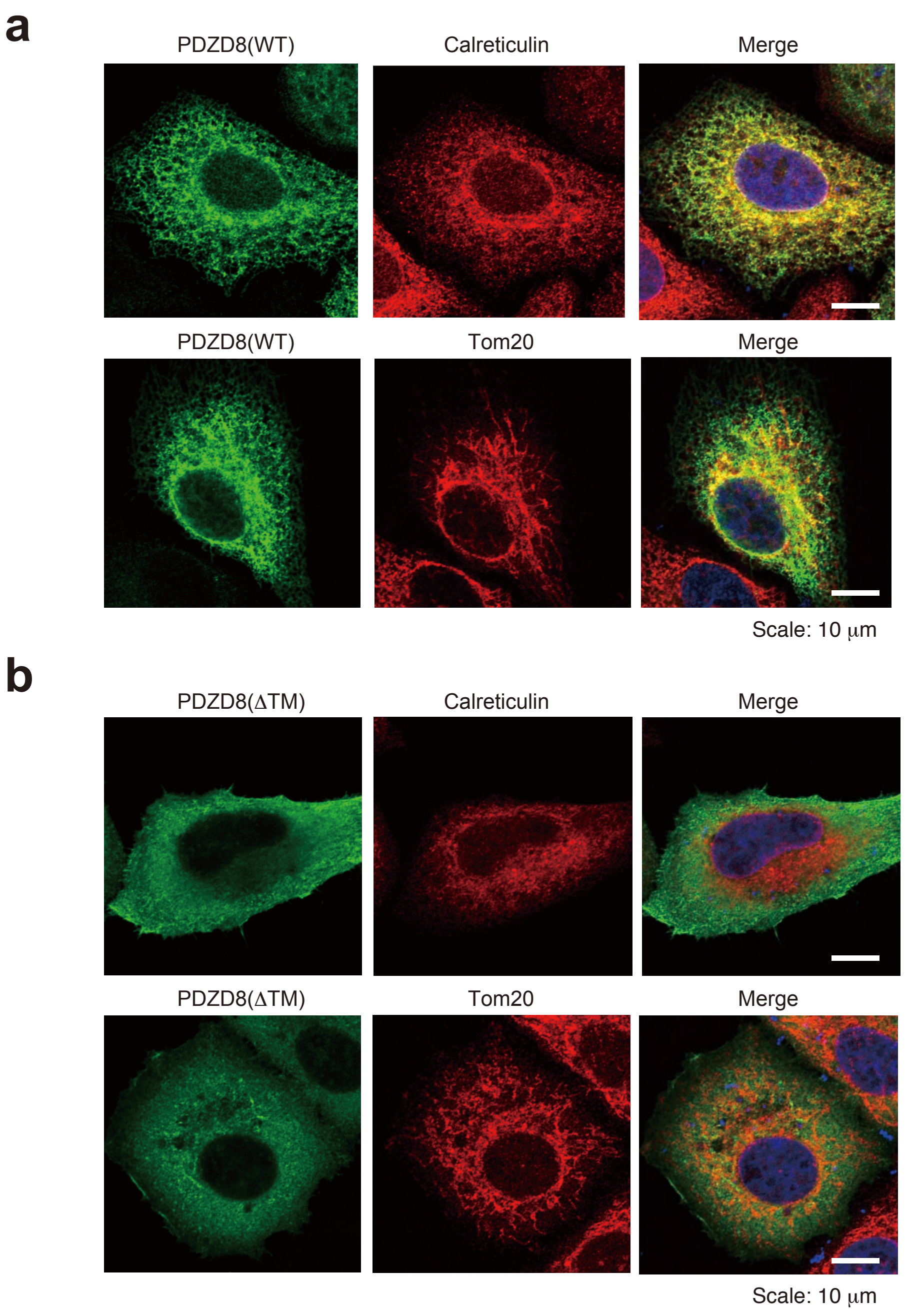

Shirane et al. Figure S6

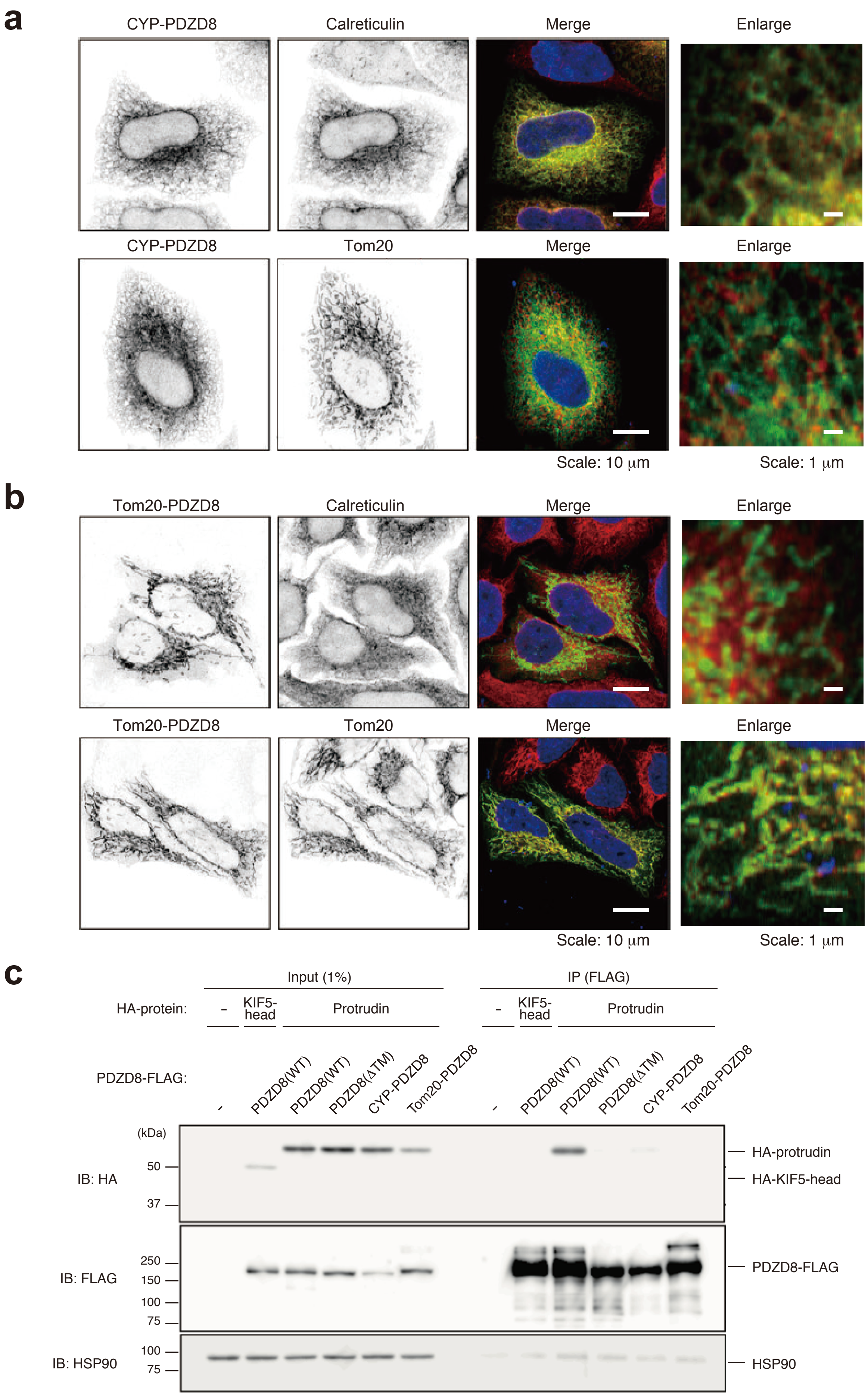

Shirane et al. Figure S7

a

Split-GFP (ER-LyLE) + LAMP1 (LyLE)　　　　Split-GFP (ER-LyLE) + Tom20 (Mito)

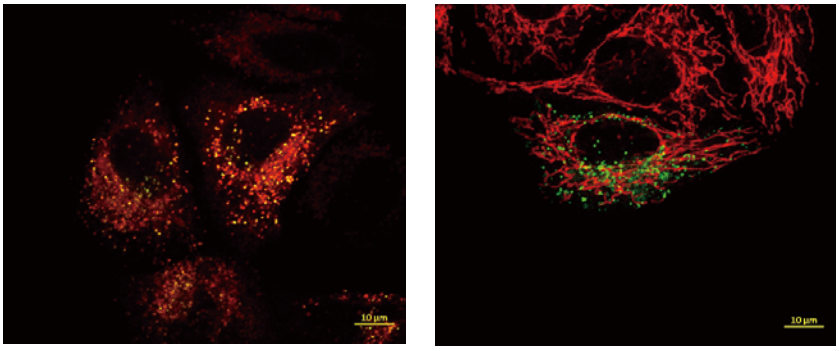

b

Split-GFP (ER-Mito) + LAMP1 (LyLE)　　　　Split-GFP (ER-Mito) + Tom20 (Mito)

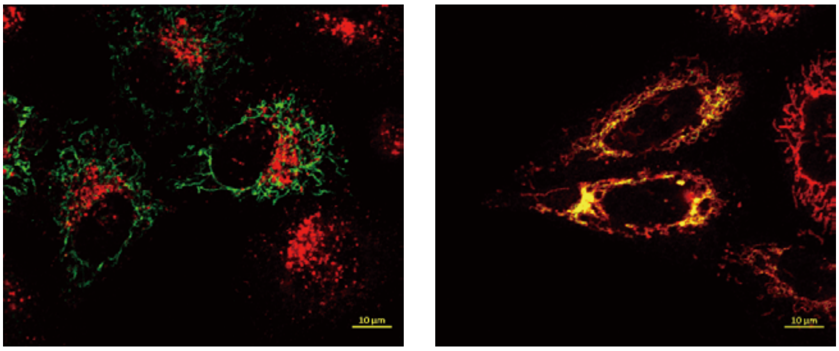

c

Split-GFP (ER-LyLE) + mCherry

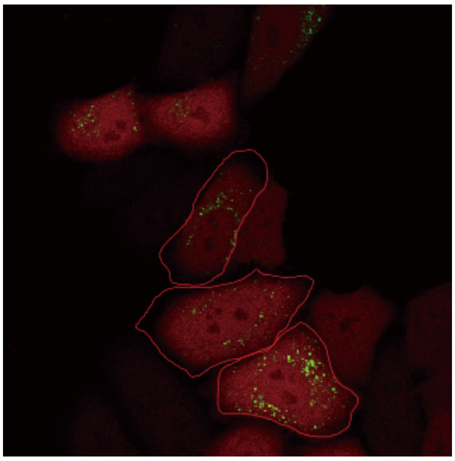

Shirane et al. Figure S8

a

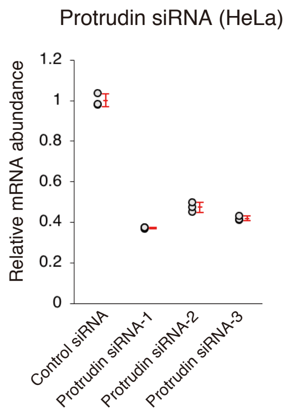

b

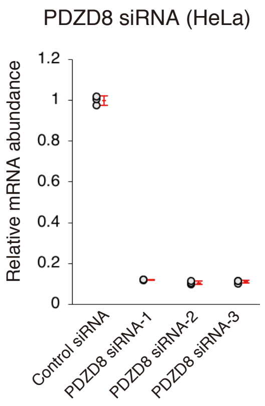

c

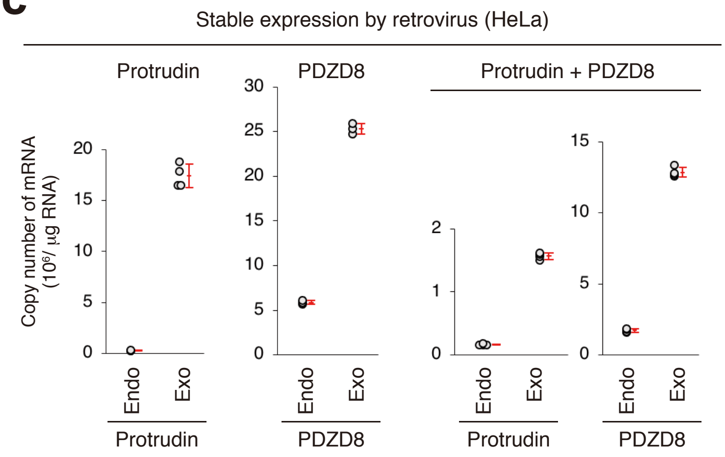

d

(10⁶/ µg RNA)

Retrovirus	mRNA	Endo/Exo	Mean	SD
Protrudin	Protrudin	Endo	0.30	0.01
		Exo	17.42	1.13
PDZD8	PDZD8	Endo	5.87	0.20
		Exo	25.33	0.57
Protrudin + PDZD8	Protrudin	Endo	0.17	0.01
		Exo	1.57	0.05
	PDZD8	Endo	1.72	0.11
		Exo	12.84	0.34

e

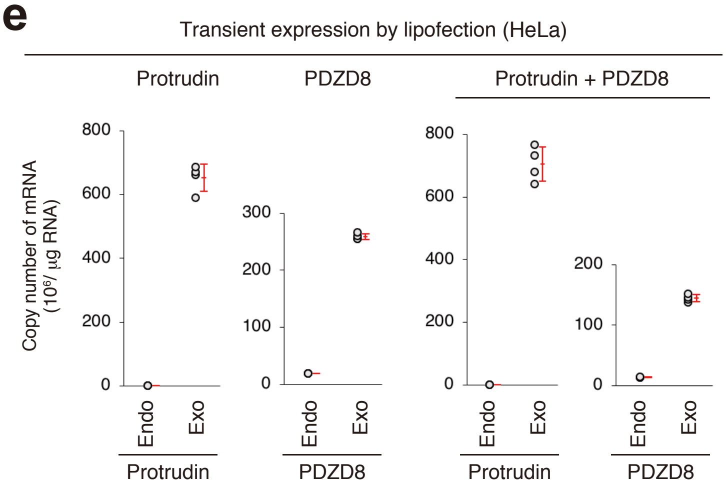

f

(10⁶/ µg RNA)

Lipofection	mRNA	Endo/Exo	Mean	SD
Protrudin	Protrudin	Endo	0.62	0.02
		Exo	652.50	42.44
PDZD8	PDZD8	Endo	19.71	0.24
		Exo	258.95	5.51
Protrudin + PDZD8	Protrudin	Endo	0.56	0.02
		Exo	705.00	55.00
	PDZD8	Endo	14.12	0.26
		Exo	144.37	6.00

g

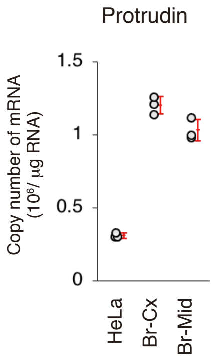

h

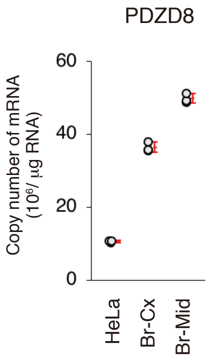

Shirane et al. Figure S9

a

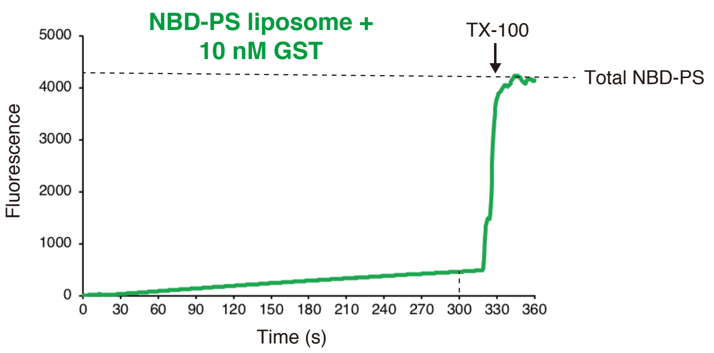

b

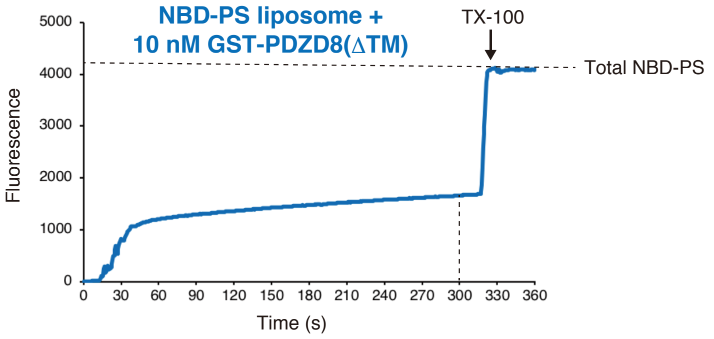

c

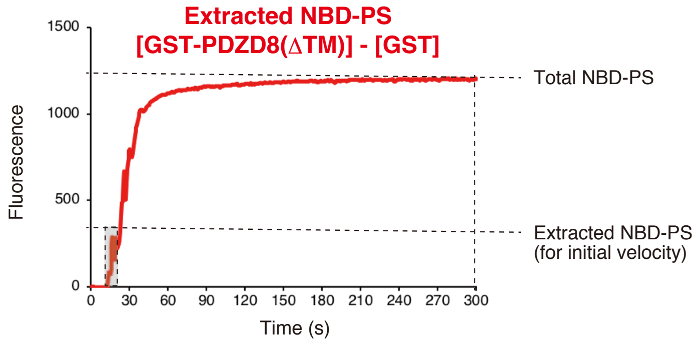

d

Fig.3e

NBD-Lipid	PA	PS	PE	PC	Cer	Chol
GST-PDZD8	ΔTM, 10 nM	ΔTM, 10 nM	ΔTM, 10 nM	ΔTM, 10 nM	ΔTM, 10 nM	ΔTM, 10 nM
Velocity (nM lipid/s)	5.89	16.21	6.55	5.93	13.41	0.33

Fig.3h

NBD-Lipid	PS (-Acc)	PS (-Acc)	PS (-Acc)	PS (+Acc)	PS (+Acc)	PS (+Acc)
GST-PDZD8	ΔTM, 5 nM	ΔTM, 10 nM	ΔTM, 20 nM	ΔTM, 5 nM	ΔTM, 10 nM	ΔTM, 20 nM
Velocity (nM lipid/s)	3.17	16.21	26.08	2.74	18.67	22.98

Fig.4c

NBD-Lipid	PS	PS	PS	PS	PS	PS	PS
GST-PDZD8 (5 nM)	ΔTM	ΔTMΔSMP	ΔTMΔPDZ	ΔTMΔPRT	ΔTMΔC1	SMP	SMP-PDZ
Velocity (nM lipid/s)	3.07	1.21	2.03	2.55	3.32	0.36	1.76

Fig.5g

NBD-Lipid	PS	PS	PS
GST-PDZD8 (10 nM)	ΔTM	ΔTMΔCC	ΔTMΔCCΔSMP
Velocity (nM lipid/s)	15.28	21.74	3.79

Fig.4f

NBD-Lipid	PS (+DGS, -Acc)	PS (+DGS,+Acc)	PS (+DGS, -Acc)	PS (+DGS,+Acc)
His-protein (10 nM)	PDZD8(ΔTM)	PDZD8(ΔTM)	GST	GST
Velocity (nM lipid/s)	0.27	0.25	0.00	0.06

Shirane et al. Figure S10

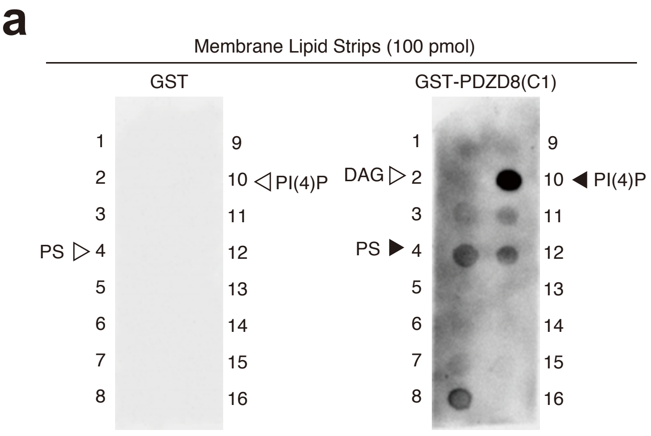

1. Glyceryl tripalmitate/triglyceride (GT)	9. Phosphatidylinositol (PI)
2. Diacylglycerol (DAG)	10. PtdIns(4)P
3. Phosphatidic acid (PA)	11. PtdIns(4,5)P_2
4. Phosphatidylserine (PS)	12. PtdIns(3,4,5)P_3
5. Phosphatidylethanolamine (PE)	13. Cholesterol
6. Phosphatidylcholine (PC)	14. Sphingomyelin (SM)
7. Phosphatidylglycerol (PG)	15. Sulfatide
8. Cardiolipin (CL)	16. Blue blank

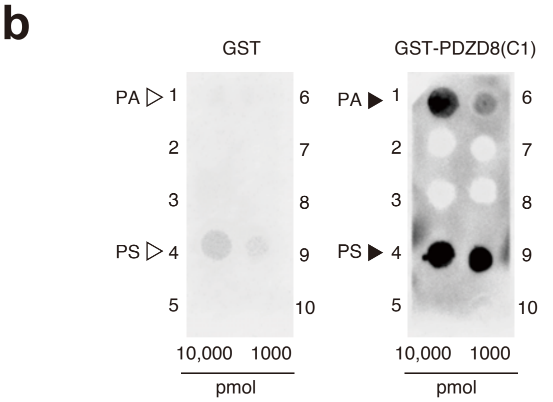

1, 6	PA
2, 7	PC
3, 8	PE
4, 9	PS
5, 10	Blank

Shirane et al. Figure S11

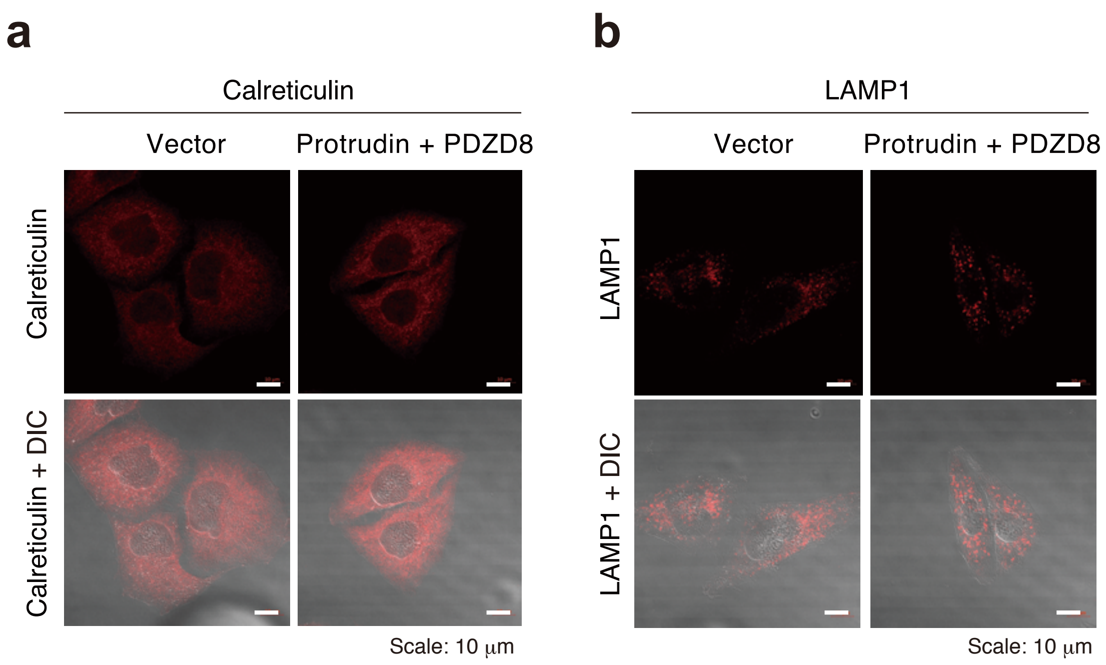

Shirane et al. Figure S12

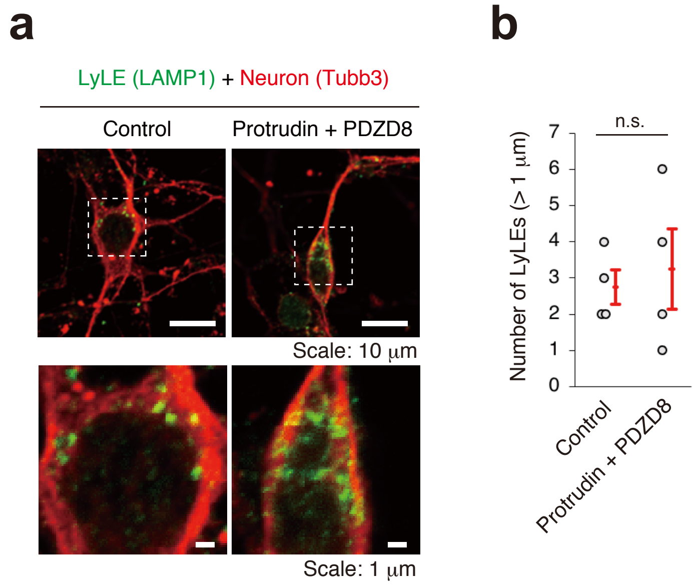

Shirane et al. Figure S13

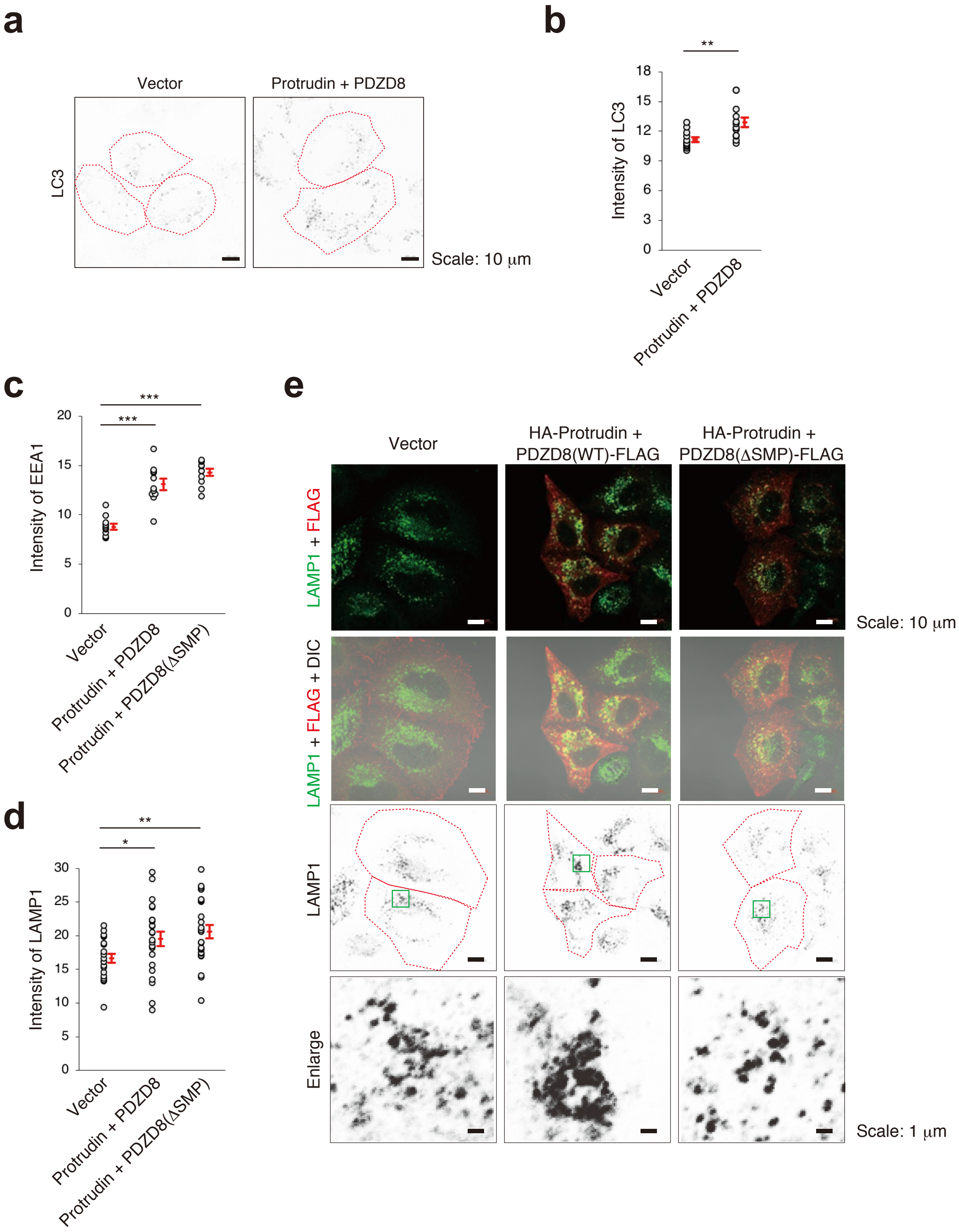

Shirane et al. Figure S14

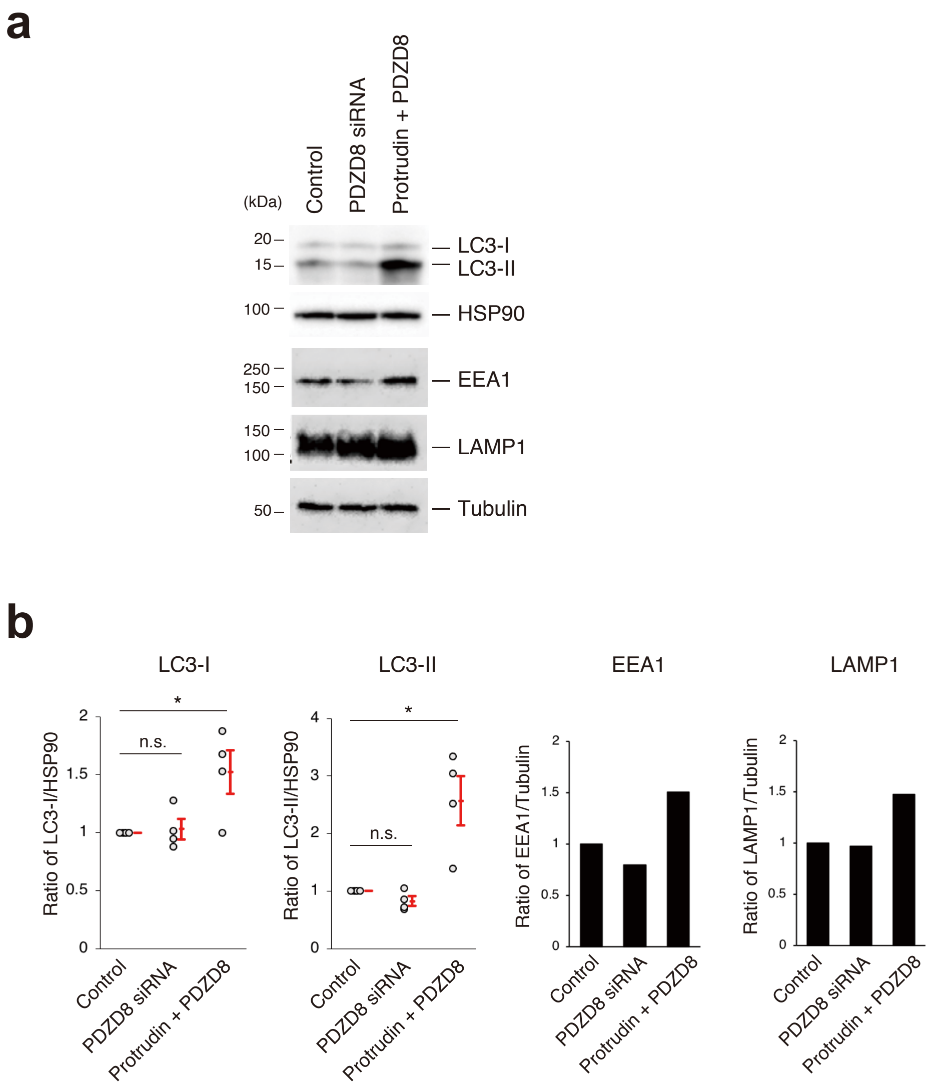

Shirane et al. Figure S15

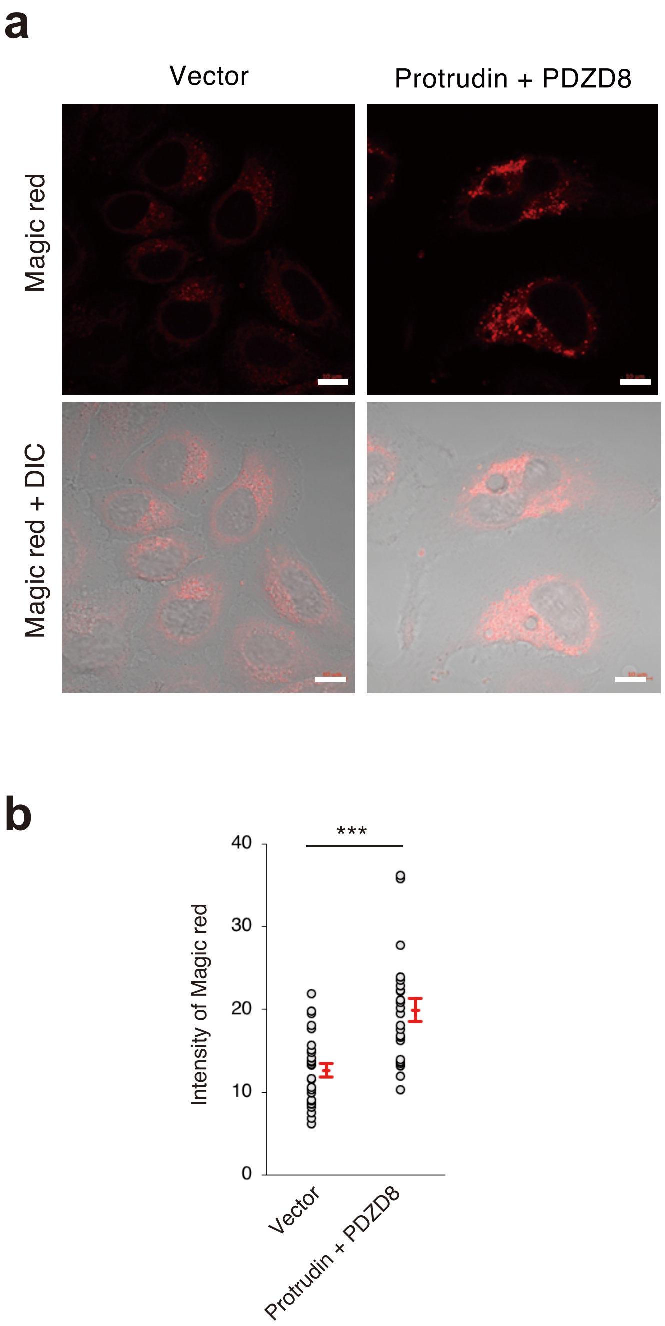

Shirane et al. Figure S16

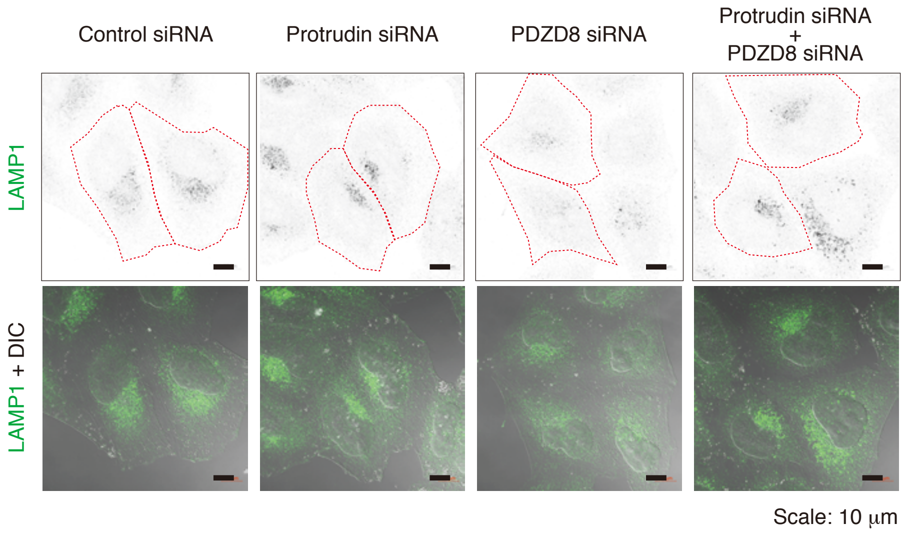

Shirane et al. Figure S17
