# Supplementary material for: Protrudin and PDZD8 contribute to neuronal integrity by promoting lipid extraction required for endosome maturation
Source: Nat Commun. 2020 Sep 11;11:4576. doi: 10.1038/s41467-020-18413-9 (PMC7486383; doi:10.1038/s41467-020-18413-9)
Supplement: Supplementary file 3 — Reporting Summary [file 41467_2020_18413_MOESM3_ESM.pdf]

natureresearch

Corresponding author(s): Keiichi I. Nakayama

Last updated by author(s): Jun 27, 2020

Reporting Summary

Nature Research wishes to improve the reproducibility of the work that we publish. This form provides structure for consistency and transparency in reporting. For further information on Nature Research policies, see Authors & Referees and the Editorial Policy Checklist .

Statistics

For all statistical analyses, confirm that the following items are present in the figure legend, table legend, main text, or Methods section.

n/a	Confirmed	
☐	☒	The exact sample size (n) for each experimental group/condition, given as a discrete number and unit of measurement
☐	☒	A statement on whether measurements were taken from distinct samples or whether the same sample was measured repeatedly
☐	☒	The statistical test(s) used AND whether they are one- or two-sided<br>*Only common tests should be described solely by name; describe more complex techniques in the Methods section.*
☒	☐	A description of all covariates tested
☐	☒	A description of any assumptions or corrections, such as tests of normality and adjustment for multiple comparisons
☐	☒	A full description of the statistical parameters including central tendency (e.g. means) or other basic estimates (e.g. regression coefficient) AND variation (e.g. standard deviation) or associated estimates of uncertainty (e.g. confidence intervals)
☐	☒	For null hypothesis testing, the test statistic (e.g. F, t, r) with confidence intervals, effect sizes, degrees of freedom and P value noted<br>*Give P values as exact values whenever suitable.*
☒	☐	For Bayesian analysis, information on the choice of priors and Markov chain Monte Carlo settings
☐	☒	For hierarchical and complex designs, identification of the appropriate level for tests and full reporting of outcomes
☐	☒	Estimates of effect sizes (e.g. Cohen's d, Pearson's r), indicating how they were calculated

Our web collection on statistics for biologists contains articles on many of the points above.

Software and code

Policy information about availability of computer code

Data collection	MS analysis: The membrane fraction of Neuro2A cells expressing His6-FLAG–tagged mouse protrudin was subjected to affinity purification with anti-FLAG (M2)–agarose affinity gel (Sigma-Aldrich), and the material eluted with FLAG peptide (Sigma-Aldrich) was then subjected to affinity purification with Ni-NTA agarose (ProBond resin, Invitrogen Life Technologies). Proteins eluted with imidazole were concentrated by precipitation with chloroform-methanol, fractionated by SDS-PAGE, and stained with silver. The membrane fraction of mouse brain was subjected to immunoprecipitation with mouse monoclonal antibodies to protrudin, and the resulting immunoprecipitates were fractionated by SDS-PAGE and stained with silver. The stained gels were sliced into pieces, and the abundant proteins therein were subjected to in-gel digestion with trypsin. The resulting peptides from Neuro2A cells and mouse brain were dried, dissolved in a mixture of 0.1% trifluoroacetic acid and 2% acetonitrile, and then applied to a nanoflow LC system (Paradigm MS4; Michrom BioResources, Auburn, CA) equipped with an L-column (C18, 0.15 by 50 mm, particle size of 3m; CERI, Tokyo, Japan). Nanoscale LC (nanoLC)–MS/MS analysis was performed with a system consisting of a Q Exactive Plus mass spectrometer (Thermo Fisher Scientific) coupled with a nanoLC instrument (Advance, Michrom BioResources).
Data analysis	All MS/MS spectra were compared with protein sequences in the International Protein Index (IPI, European Bioinformatics Institute) mouse version 3.44 with the use of the MASCOT algorithm. Assigned high-scoring peptide sequences (MASCOT score of ≥35) were considered for correct identification. Identified peptides from independent experiments were integrated and regrouped by IPI accession number. For the mouse brain experiments, the peptides identified in protrudin knockout mice were subtracted from those identified in WT mice.

For manuscripts utilizing custom algorithms or software that are central to the research but not yet described in published literature, software must be made available to editors/reviewers. We strongly encourage code deposition in a community repository (e.g. GitHub). See the Nature Research guidelines for submitting code & software for further information.

Data

Policy information about availability of data

All manuscripts must include a data availability statement. This statement should provide the following information, where applicable:

- Accession codes, unique identifiers, or web links for publicly available datasets
- A list of figures that have associated raw data
- A description of any restrictions on data availability

The MS data have been deposited in the J-POST Repository Database

PRT complex in mouse brain, WT
ID: PXD016817
URL: https://repository.jpostdb.org/preview/19895863135dfb1f7c74458
Access key: 3055

PRT complex in mouse brain, KO
ID: PXD016818
URL: https://repository.jpostdb.org/preview/18913954415dfb1f7911eb8
Access key: 5957

Field-specific reporting

Please select the one below that is the best fit for your research. If you are not sure, read the appropriate sections before making your selection.

☒ Life sciences ☐ Behavioural & social sciences ☐ Ecological, evolutionary & environmental sciences

For a reference copy of the document with all sections, see nature.com/documents/nr-reporting-summary-flat.pdf

Life sciences study design

All studies must disclose on these points even when the disclosure is negative.

Sample size	*Describe how sample size was determined, detailing any statistical methods used to predetermine sample size OR if no sample-size calculation was performed, describe how sample sizes were chosen and provide a rationale for why these sample sizes are sufficient.*
Data exclusions	*Describe any data exclusions. If no data were excluded from the analyses, state so OR if data were excluded, describe the exclusions and the rationale behind them, indicating whether exclusion criteria were pre-established.*
Replication	*Describe the measures taken to verify the reproducibility of the experimental findings. If all attempts at replication were successful, confirm this OR if there are any findings that were not replicated or cannot be reproduced, note this and describe why.*
Randomization	*Describe how samples/organisms/participants were allocated into experimental groups. If allocation was not random, describe how covariates were controlled OR if this is not relevant to your study, explain why.*
Blinding	*Describe whether the investigators were blinded to group allocation during data collection and/or analysis. If blinding was not possible, describe why OR explain why blinding was not relevant to your study.*

Reporting for specific materials, systems and methods

We require information from authors about some types of materials, experimental systems and methods used in many studies. Here, indicate whether each material, system or method listed is relevant to your study. If you are not sure if a list item applies to your research, read the appropriate section before selecting a response.

Materials & experimental systems

n/a	Involved in the study	
☐	☒	Antibodies
☐	☒	Eukaryotic cell lines
☒	☐	Palaeontology
☐	☒	Animals and other organisms
☒	☐	Human research participants
☒	☐	Clinical data

Methods

n/a	Involved in the study	
☒	☐	ChIP-seq
☒	☐	Flow cytometry
☒	☐	MRI-based neuroimaging

Antibodies

Antibodies used	For proteomics analysis and other immunoprecipitation, a mouse monoclonal antibody to protrudin was generated by Kurabo (Osaka, Japan) with the use of a hexahistidine (His6)–tagged recombinant protein comprising amino acids 212 to 323 of mouse protrudin-L22 that had been expressed in and purified from Escherichia coli. Antibodies to protrudin (for immunoblot analysis) and to TMEM55B were obtained from ProteinTech (Chicago, IL); those to HSP90 were from BD Biosciences (San Jose, CA); those to FLAG (mouse monoclonal M2 for immunoprecipitation, immunoblot, and immunofluorescence analyses and rabbit polyclonal for immunoblot analysis), to the Myc epitope (9E10), to Tau1, and to Tubb3 were from Sigma-Aldrich; those to HA (HA.11) were from Covance (Princeton, NJ); those to calreticulin were from Stressgen (Victoria, British Columbia, Canada); those to PDZD8 were from LSBio (Seattle, WA); those to Rab7, to Rab9, to LC3, and to LAMP1 (for HeLa cells) were from Cell Signaling Technology (Beverly, MA); those to GST were from Frontier Institute (Hokkaido, Japan); those to HA (Y-11), to Tom20, and to LAMP1 (for neurons) were from Santa Cruz Biotechnology (Santa Cruz, CA); those to Map2 were from Abcam (Cambridge, MA); those to calnexin were from Enzo Life Sciences (Farmingdale, NY); those to-tubulin (TU01) were from Thermo Fisher Scientific (Waltham, MA); and those to EEA1 were from BD Transduction Laboratories (Franklin Lakes, NJ). Alexa Fluor 488– or Alexa Fluor 546–conjugated goat antibodies to mouse or rabbit immunoglobulin G (IgG) were obtained from Molecular Probes (Eugene, OR).
Validation	A mouse monoclonal antibody to protrudin was validated on its specificity by using brain extracts from wild type and protrudin deficient mice.

Eukaryotic cell lines

Policy information about cell lines

Cell line source(s)	Neuro2A, HEK293T, HeLa, and PC12 were obtained from ATCC (American Type Culture Collection). Retrovirus packaging cell line Plat-E cells were obtained from T. Kitamura (PMID: 10871756 DOI: 10.1038/sj.gt.3301206).
Authentication	*Describe the authentication procedures for each cell line used OR declare that none of the cell lines used were authenticated.*
Mycoplasma contamination	Cells were tested for mycoplasma using the MC-210 (KAC, Cat #88101-2) and PCR analysis. All cells were confirmed to be free of mycoplasma.
Commonly misidentified lines (See ICLAC register)	*Name any commonly misidentified cell lines used in the study and provide a rationale for their use.*

Animals and other organisms

Policy information about studies involving animals; ARRIVE guidelines recommended for reporting animal research

Laboratory animals	C57BL/6J and Jcl-ICR mice were obtained from CLEA Japan.
Wild animals	*Provide details on animals observed in or captured in the field; report species, sex and age where possible. Describe how animals were caught and transported and what happened to captive animals after the study (if killed, explain why and describe method; if released, say where and when) OR state that the study did not involve wild animals.*
Field-collected samples	*For laboratory work with field-collected samples, describe all relevant parameters such as housing, maintenance, temperature, photoperiod and end-of-experiment protocol OR state that the study did not involve samples collected from the field.*
Ethics oversight	*Identify the organization(s) that approved or provided guidance on the study protocol, OR state that no ethical approval or guidance was required and explain why not.*

Note that full information on the approval of the study protocol must also be provided in the manuscript.
